# Supplementary material for: CTCF and transcription influence chromatin structure re-configuration after mitosis
Source: Nat Commun. 2021 Aug 27;12:5157. doi: 10.1038/s41467-021-25418-5 (PMC8397779; doi:10.1038/s41467-021-25418-5)
Supplement: Supplementary file 1 — Supplementary information [file 41467_2021_25418_MOESM1_ESM.pdf]

Supplementary information

CTCF and transcription influence chromatin structure re-configuration after mitosis

Haoyue Zhang[1,2*], Jessica Lam[2,3], Di Zhang[2,3], Yemin Lan[3], Marit W. Vermunt[2], Cheryl A. Keller[4], Belinda Giardine[4], Ross C. Hardison[4] and Gerd A. Blobel[2,3,*]

[1]Institute of Molecular Physiology, Shenzhen Bay Laboratory, Shenzhen, Guangdong, China.

[2]Division of Hematology, The Children's Hospital of Philadelphia, Philadelphia, PA, USA

[3]Perelman School of Medicine, University of Pennsylvania, Philadelphia, PA, USA

[4]Department of Biochemistry and Molecular Biology, Pennsylvania State University, University Park, PA, USA

*Correspondence: zhang_adam@szbl.ac.cn (H.Z.), blobel@email.chop.edu (G.A.B)

Supplementary Figure 1

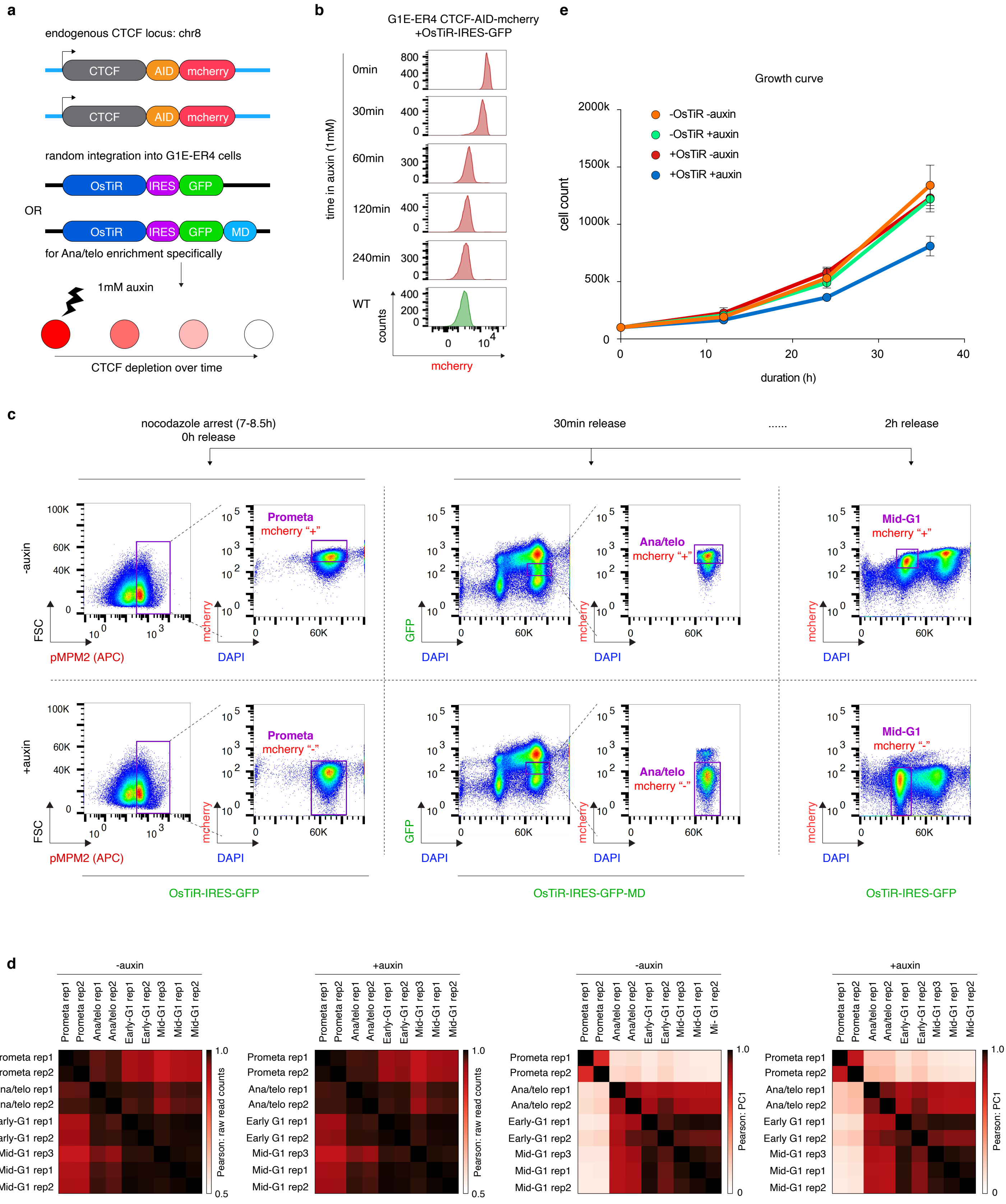

Supplementary Figure 1

a, Schematic showing the construction of G1E-ER4 CTCF-AID-mCherry cell line and ectopic expression of Os-TIR-IRES-GFP. Os-TIR-IRES-GFP was used for prometaphase, early- and mid-G1 phase. To enable enrichment of ana/telophase cells, we specifically over expressed Os-TiR-IRES-GFP-MD. MD denotes mitotic degron. **b**, Flow cytometry plot showing the acute depletion of mCherry signal in asynchronous cells upon auxin treatment. Flow plots are representative of two independent experiments. **c**, FACS plots and gates (purple boxes) used for purification of mitotic and post-mitotic populations with and without CTCF. One set of plots representative of two independent biological replicates is show. **d**, Heatmaps showing Pearson correlations among Hi-C samples based on 100kb binned raw read counts and eigenvector 1 values, respectively. Note that samples with or without auxin treatment were separately plotted. **e**, Growth curves showing cell proliferation with or without auxin treatment. Note, cells devoid of OsTiR were included as controls. Error bar denote SEM (n=3 biological replicates). Source data are provided as a Source Data file.

Supplementary Figure 2

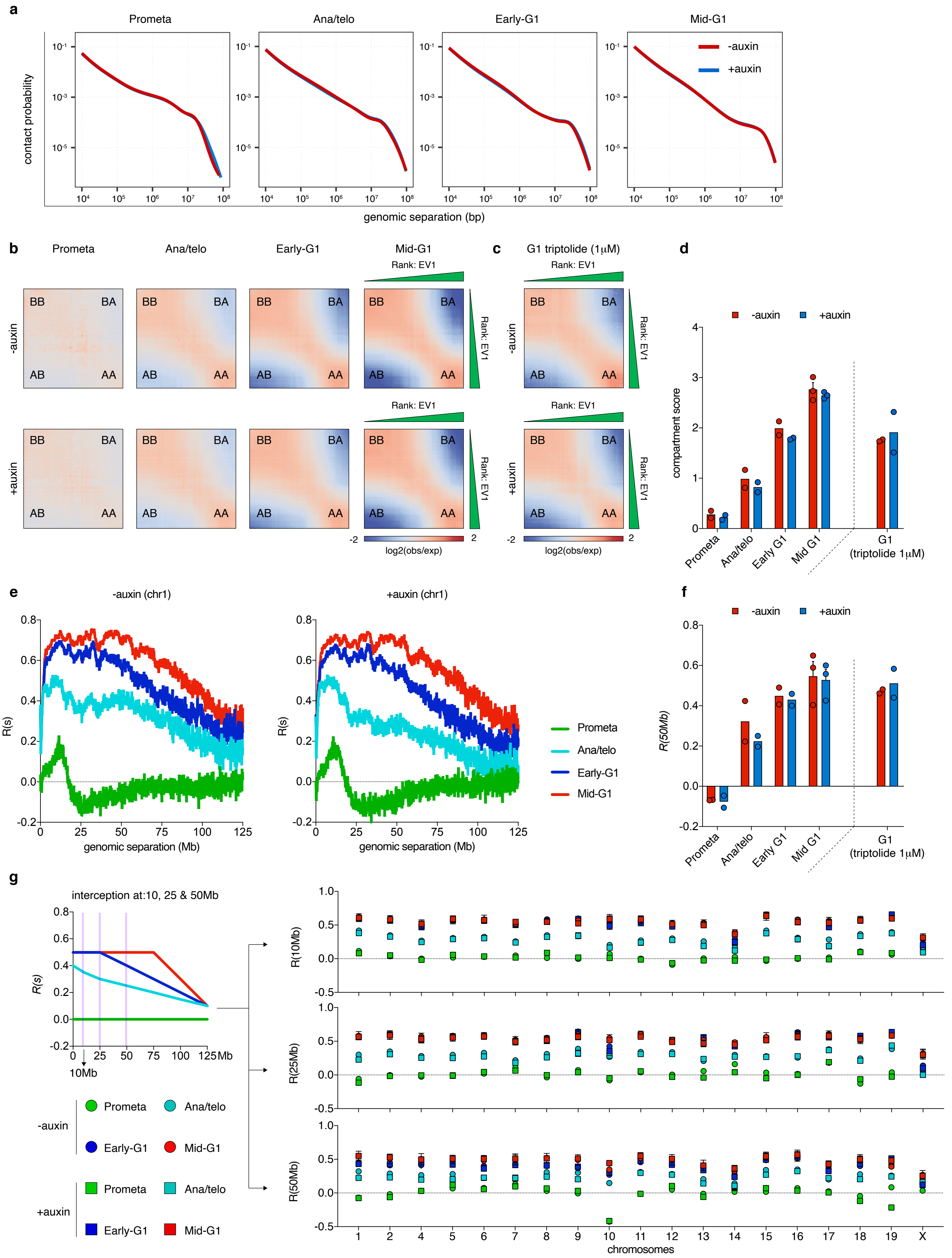

Supplementary Figure 2

a, Chromosome averaged distance dependent contact frequency decay curves across cell cycle stages in control and auxin treated samples. **b**, Saddle plots showing compartment strengths across cell cycle stages in untreated and auxin treated samples. **c**, Saddle plots showing compartment strength in G1 phase cells after triptolide treatment. **d**, Bar graphs showing compartment scores for each cell cycle stage in untreated and auxin treated samples. n=2 biological replicates for prometa, and/telo and early-G1 samples and n=3 for mid-G1 samples either with or without auxin treatment. Compartment scores of triptolide treated G1 samples (n=2 biological replicates either with or without auxin treatment) were plotted on the right. Error bars of mid-G1 samples represent SEM. **e**, Line graphs showing the level of compartmentalization *R* vs. genomic separations *s* for chromosome 1 for each cell cycle stage in both untreated and auxin treated samples. The gradual flattening of curves as cells progress towards G1 suggests expansion of the plaid-like compartmental interaction patterns from diagonal proximal regions to diagonal distal regions. **f**, Bar graphs showing the level of compartmentalization at 50Mb *R(50Mb)* for each cell cycle stage with or without CTCF. n=2 biological replicates for prometa, and/telo and early-G1 samples and n=3 for mid-G1 samples. *R(50Mb)* of the triptolide treated G1 samples (n=2 biological replicates either with or without auxin treatment) were plotted on the right. Error bars of mid-G1 samples represent SEM. **g**, Left: Cartoon line plot of *R(s)* across different time points showing a series of intersections at 10Mb, 25Mb and 50Mb. Right: Replicate averaged *R(s)* of each individual chromosome across all cell cycle stages in both untreated and auxin treated samples when *s* equals to 10Mb, 25Mb and 50Mb respectively. Error bars denote SEM for mid-G1 samples (n=3 biological replicates).

Supplementary Figure 3

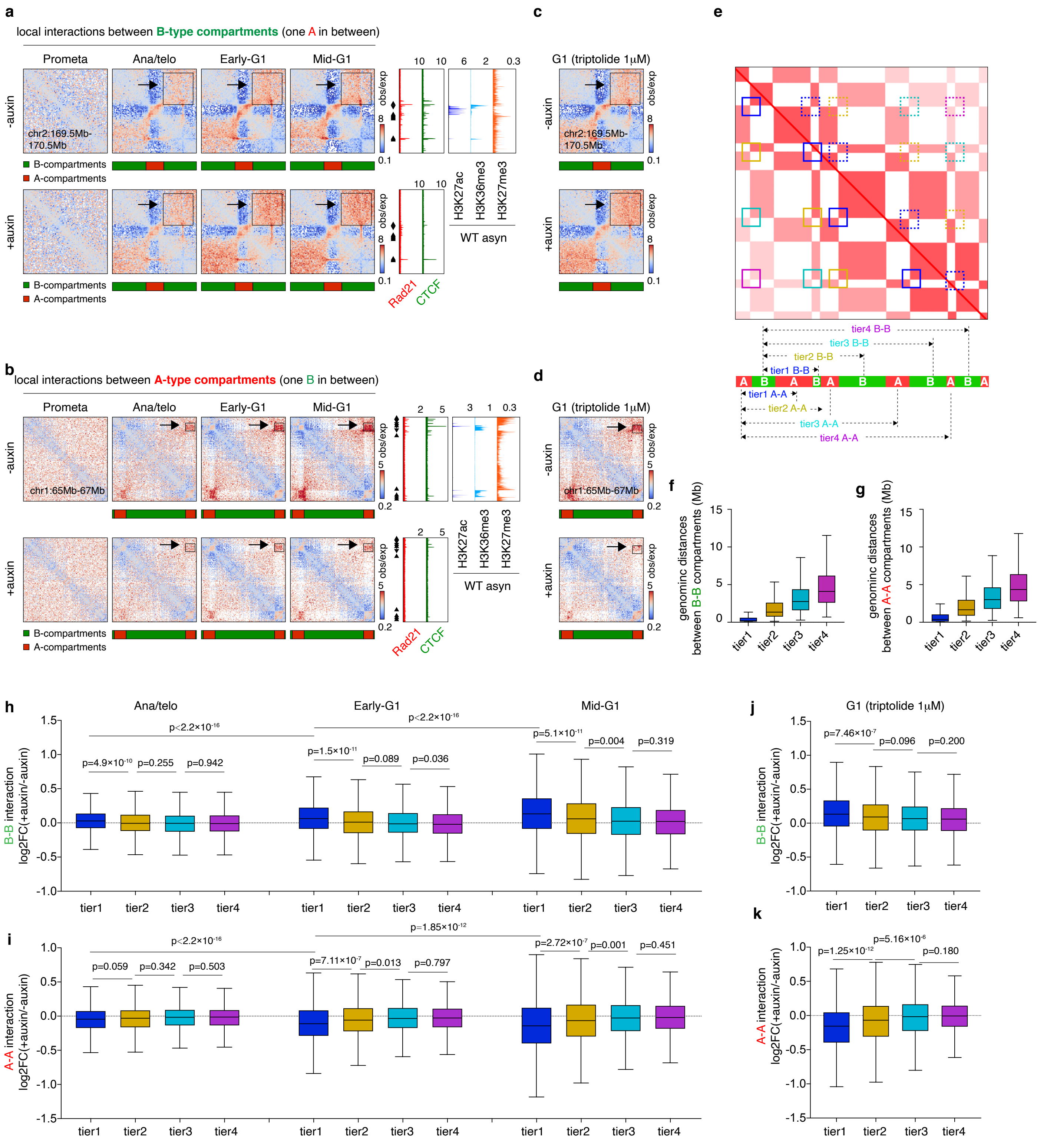

Supplementary Figure 3

a, Additional example of enhanced local B-B interactions after CTCF depletion. Bin size: 10kb. Arrows and boxes highlight the increased local B-B interactions after CTCF depletion across post-mitotic cell cycle stages. Tracks of CTCF and Rad21 with or without auxin treatment as well as H3K27ac, H3K36me3 and H27me3 are from asynchronously growing G1E-ER4 cells. **b**, Additional example of reduced local A-A interactions after CTCF depletion. **c**, KR balanced Hi-C contact matrices showing the same region as in (**a**) in G1 phase cells after triptolide treatment. **d**, KR balanced Hi-C contact matrices showing the same region as (**b**) in G1 phase cells after triptolide treatment. **e**, Schematic of the checkerboard pattern of compartments. Tier1-4 B-B interactions denote contacts between B-type compartments interspersed with 1-4 A-type compartments and are demarcated by dotted boxes. Tier1-4 A-A interactions denote contacts between A-type compartments interspersed with 1-4 B-type compartments and are demarcated by solidly lined boxes. **f and g**, Boxplots showing the distances between B-B or A-A interactions, respectively from different tiers. n=1827, 1807, 1787, 1767 for tier1, 2, 3 and 4 B-B pairs respectively and n=1846, 1826, 1806, 1786 for tier 1, 2, 3 and 4 A-A pairs respectively. Boxplots present upper and lower quartiles with center line as median. Whiskers denote 1.5 × interquartile range (IQR). **h**, Boxplots showing the $\log_2$ fold change of B-B interactions from different tiers (n=1604, 1543, 1465 and 1415 for tier 1-4 B-B interactions respectively), upon CTCF loss across post-mitotic time points. P values were computed through two-sided Wilcoxon signed-rank test. Comparisons between tier1 B-B interactions across cell cycle stages suggest the progressively amplified CTCF depletion induced gains of B-B interactions after mitosis (P values calculated through two-sided paired Wilcoxon signed-rank test). Boxplots present upper and lower quartiles with center line as median. Whiskers denote 1.5 × interquartile range (IQR). **i**, Similar to (**h**),

displayed are $\log_2$ fold changes of A-A interactions from different tiers, upon CTCF loss. n=1612, 1554, 1497 and 1439 for tier1-4 A-A interactions respectively. **j**, Boxplots showing the $\log_2$ fold change of B-B interactions from different tiers upon CTCF loss after triptolide treatment. n=1604, 1543, 1465 and 1415 for tier 1-4 B-B interactions respectively. P values were computed through two-sided Wilcoxon signed-rank test. Boxplots present upper and lower quartiles with center line as median. Whiskers denote 1.5 × interquartile range (IQR). **k**, Similar to (**j**), shown are $\log_2$ fold changes of A-A interactions from different tiers upon CTCF loss after triptolide treatment. n=1612, 1554, 1497 and 1439 for tier1-4 A-A interactions respectively

Supplementary Figure 4

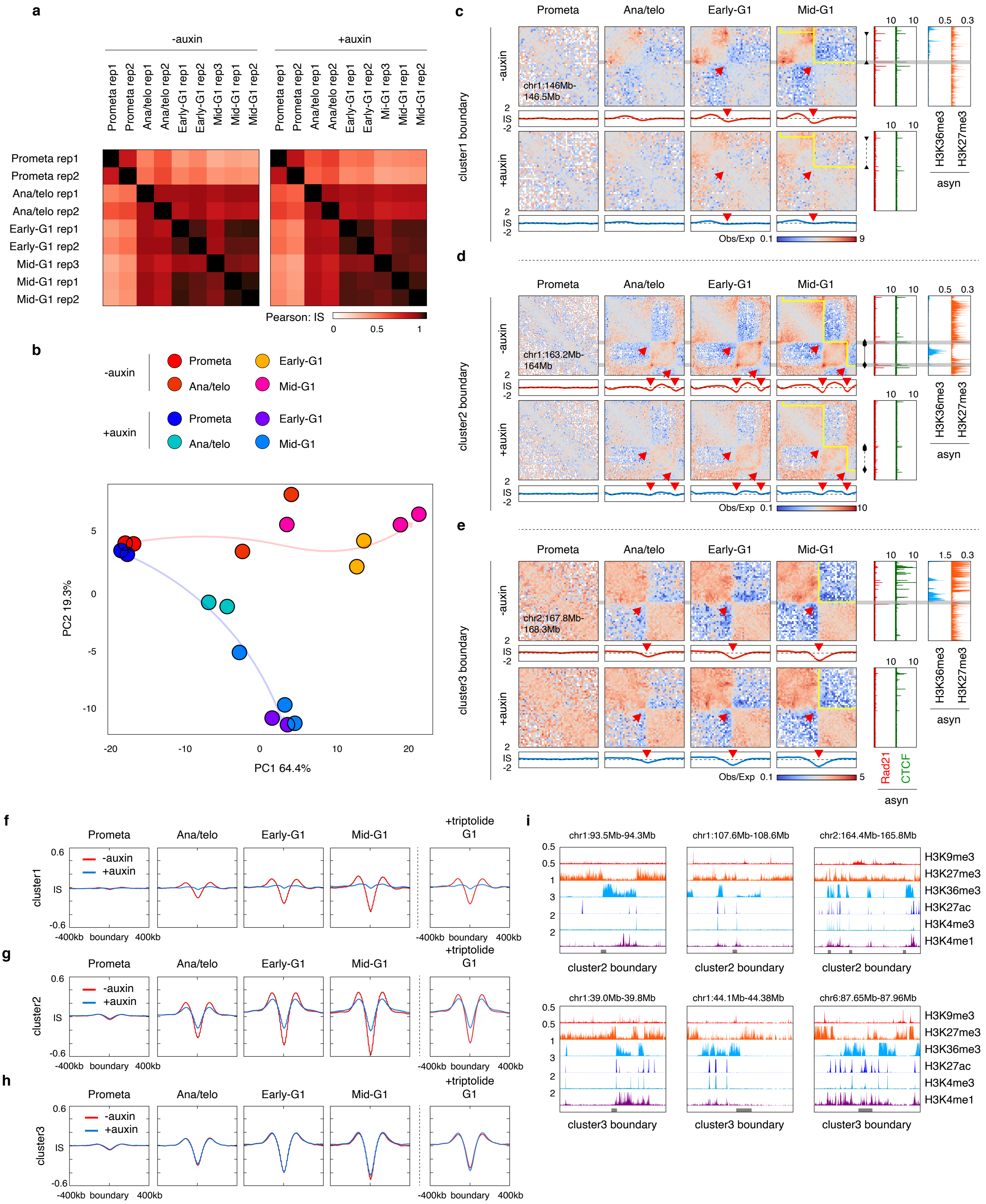

Supplementary Figure 4 | Characterization of boundary re-establishment after mitosis upon CTCF loss.

a, Pearson correlations of boundary insulation scores between biological replicates in untreated or auxin treated samples. **b**, PCA analysis of the post-mitotic boundary reformation trajectories of with or without CTCF depletion. **c**, KR balanced Hi-C contact matrices and corresponding insulation score tracks of a representative region containing a cluster1 boundary across cell cycle stages in control and auxin treated samples. Bin size: 10kb. Read arrows indicate domain boundaries. Yellow lines denote TADs identified by rGMAP. Tracks of CTCF and Rad21 with or without auxin treatment as well as H3K36me3 and H27me3 are from asynchronously growing G1E-ER4 cells. Black arrow heads denote CTCF motif orientation, and dotted line demarcates loss of loop upon auxin treatment. **d & e**, Similar to (**c**), KR balanced Hi-C contact matrices showing representative regions containing cluster2 and 3 boundaries respectively. **f-h**, Left panel: Meta-region plots of insulation score profiles centered on cluster1, 2 or 3 boundaries, across cell cycle stages in control and auxin treated samples. Right panel: Meta-region plots of insulation score profiles similar to the left in G1 samples after triptolide treatment. **i**, genome browser tracks showing the local histone modification profiles of representative cluster2 and 3 boundaries.

Supplementary Figure 5

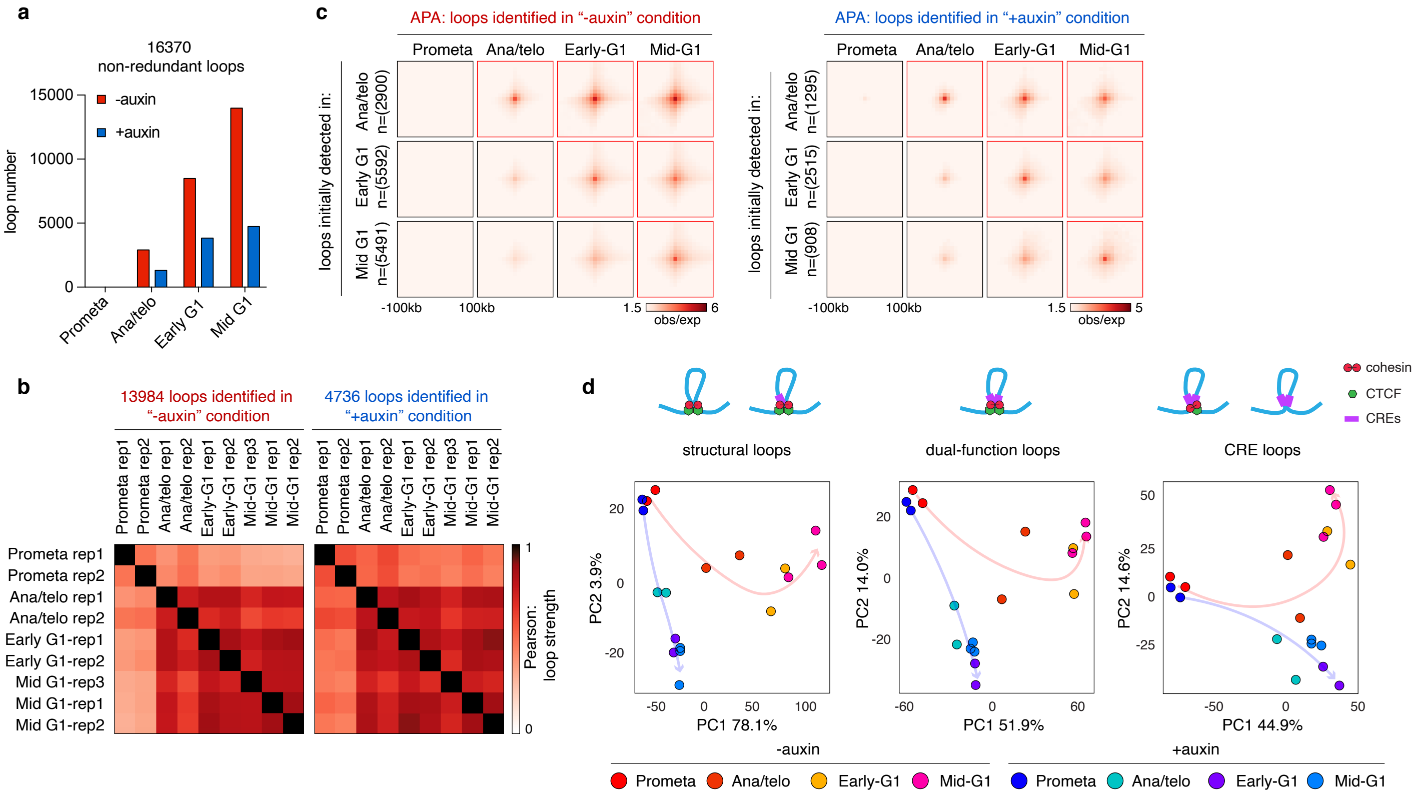

Supplementary Figure 5

a, Bar graphs showing the number of loops called at each cell cycle stage in both untreated samples and auxin treated samples. **b**, Heatmaps showing the Pearson correlation of loops called in untreated and auxin treated samples, respectively. Pearson correlations were computed based on loop strength (obs/exp). **c**, Aggregated peak analysis (APA) plots showing time dependent emergence of loops called at each cell cycle stage in untreated and auxin treated samples. Bin size: 10kb. Notably, weak APA signals were observable before the loops were called, likely due to thresholding by the loop calling algorithm. **d**, PCA analysis showing the reformation trajectories of structural loops (left), dual-function loops (middle) and CRE loops (right) respectively, in untreated as well as auxin treated samples. CRE denotes cis-regulatory elements.

Supplementary Figure 6

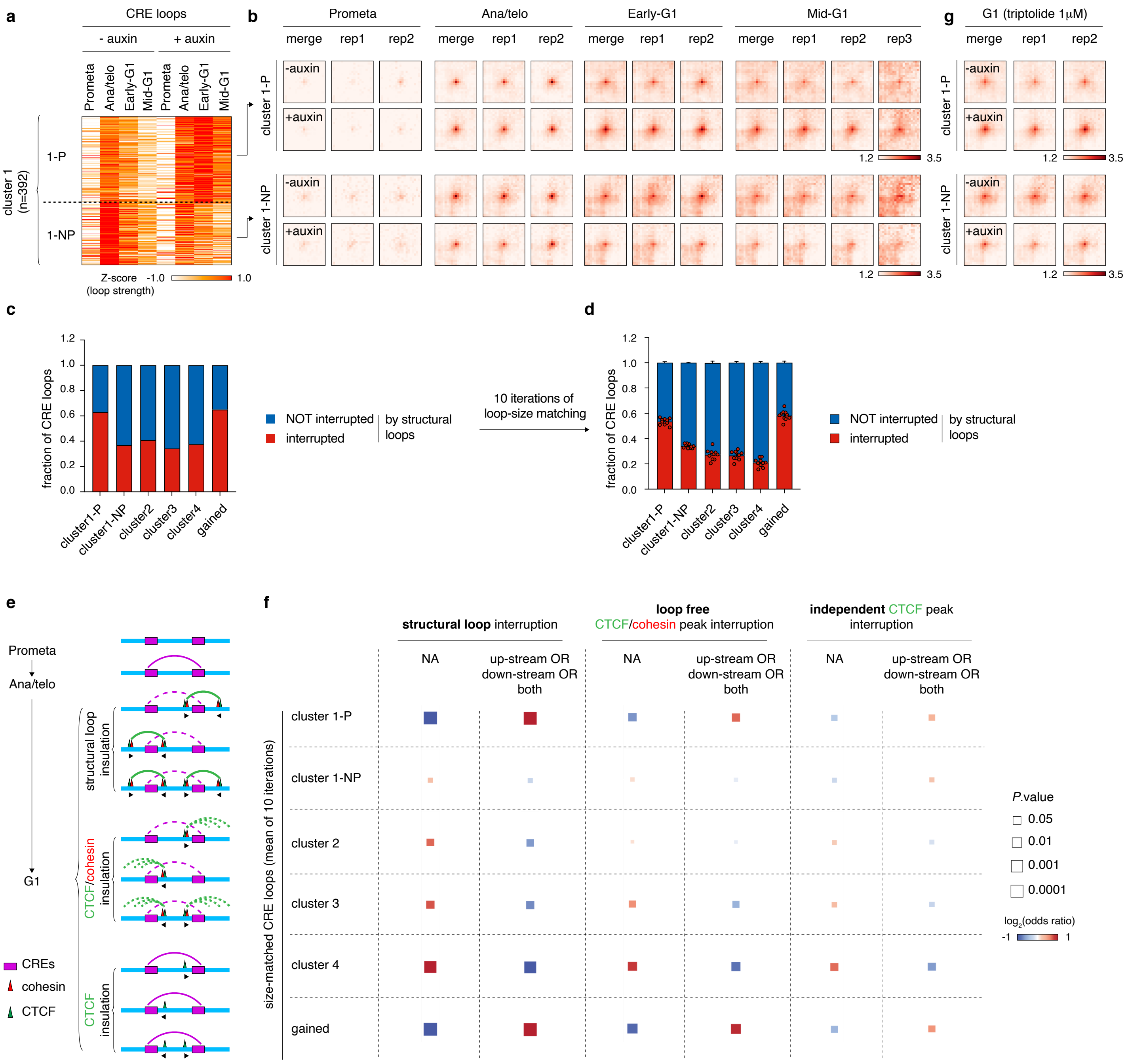

Supplementary Figure 6

a, Heatmaps showing the sub-clustering of cluster1-P (persistent upon CTCF depletion) and cluster 1-NP (non-persistent upon CTCF depletion). **b**, APA plots of loop clusters from (**a**). Bin size: 10kb. Plots for the replicate-merged as well as each individual replicates are shown. **c**, Bar graphs showing the fractions of CRE loops from indicated clusters that were interrupted by structural loops. **d**, Bar graphs depicting the fraction of size-matched CRE loops from indicated clusters that were interrupted by structural loops. Error bars denote the SEM of n=10 iterations of random size-matching operations. **e**, Schematic showing potential mechanisms (structural loops, CTCF/cohesin loop extrusion, and loop-independent CTCF) through which CTCF may exert its insulation function to disrupt transient post-mitotic CRE loops. **f**, Enrichment analysis of each sub-cluster of CRE loops controlled by mechanisms in (**e**). Left: relative enrichment analysis of whether CRE loops from each sub-cluster were interrupted by structural loops. Colors of the squares indicate $\log_2$ transformed odds ratio (two-sided Fisher's exact test). Sizes of squares indicate the significance of enrichment (*p* value from Fisher's exact test). Middle: similar to the left, showing relative enrichment of CRE loops from each sub-cluster to be interrupted by loop-free CTCF/cohesin co-occupied sites. Right: relative enrichment of CRE loops from each sub-cluster interrupted by cohesin and structural loop-independent CTCF peaks. **g**, APA plots showing the same pile-up Hi-C matrices of each CRE loop sub-cluster as (**a**, **b**), in G1 cells after triptolide treatment. Bin size: 10kb. CRE denotes cis-regulatory elements

Supplementary Figure 7

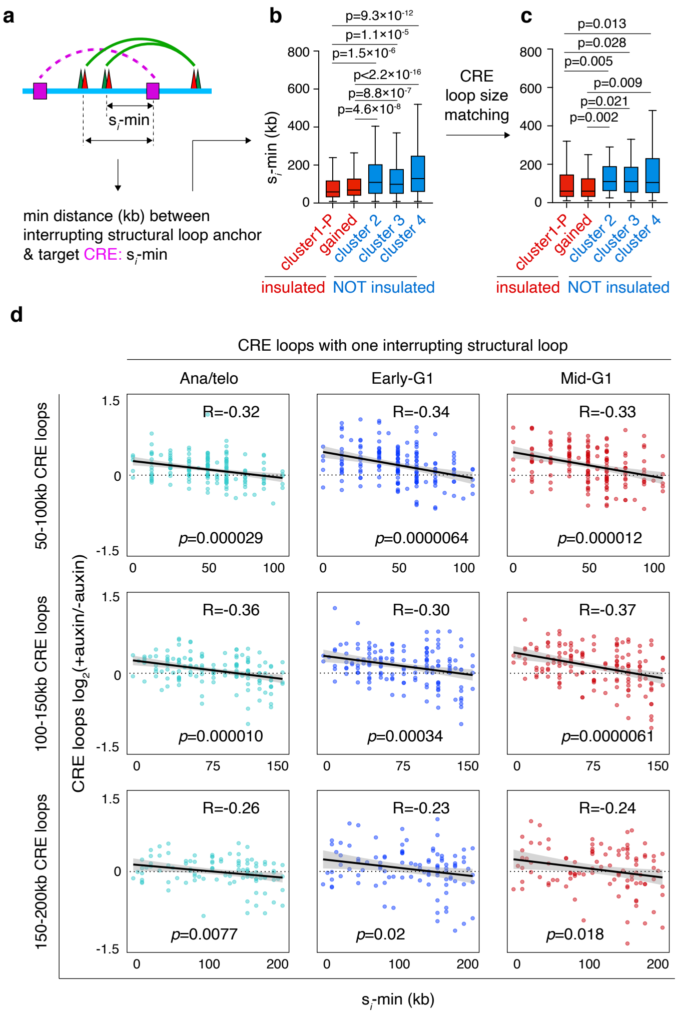

Supplementary Figure. 7

a, Definition of s_i-min. **b**, Boxplots showing significantly lower s_i-min in the insulated cluster1-P (n=144) and newly gained CRE loops (n=918) in comparison to the less insulated cluster2 (n=303), 3 (n=350) and 4 (n=408) CRE loops. CRE denotes cis-regulatory elements. P values were computed through two-sided Wilcoxon signed-rank test. Boxplots present upper and lower quartiles with center line as median. Whiskers denote 1.5 × interquartile range (IQR). **c**, Boxplots showing the same comparison as (**b**) after CRE loop size-matching. n= 49 loops for all groups. P values were computed through two-sided Wilcoxon signed-rank test. Boxplots present upper and lower quartiles with center line as median. Whiskers denote 1.5 × interquartile range (IQR). **d**, Scatter plots showing the significant negative correlation across all post-mitotic cell cycle stages between insulation strength (log_2 fold change +auxin/-auxin) and s_i-min for CRE loops with a single interrupting structural loop. Top panel: sizes of CRE loops were limited to 50-100kb. Middle panel: sizes of CRE loops were limited to 100-150kb. Bottom panel: CRE loop sizes were limited to 150-200kb. The correlation coefficient and *p* values were computed via the "cor.test" function in R. Error bands denote 95% confidence interval.

Supplementary Figure 8

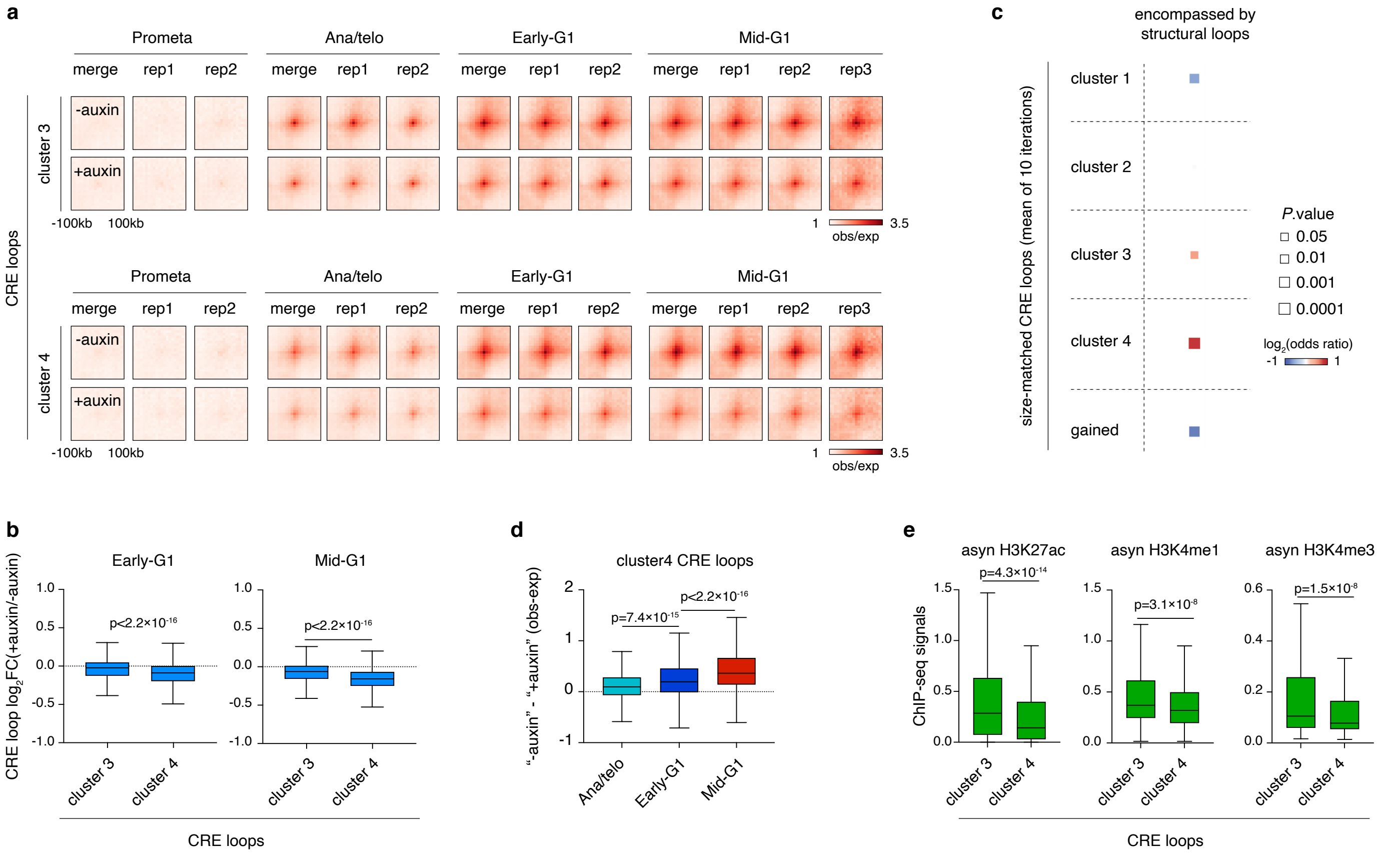

Supplementary Figure. 8

a, APA plots of the cluster3 and cluster4 CRE loops with or without auxin at all tested time points. Bin size: 10kb. Plots for the replicate-merged as well as each individual replicates are shown. **b**, Box plots showing the log_2 fold change of cluster3 (n=1019) and cluster4 (n=1081) CRE loop strength after auxin treatment in early- and mid-G1 phase. CRE denotes cis-regulatory elements. P values were computed through two-sided Wilcoxon signed-rank test. Boxplots present upper and lower quartiles with center line as median. Whiskers denote 1.5 × interquartile range (IQR). **c**, Enrichment analysis of each CRE loop cluster encompassed by structural loops. Colors of the squares indicate log_2 transformed odds ratio (two-sided Fisher's exact test). Sizes of squares indicate the significance of enrichment (P values of two-sided Fisher's exact test). **d**, Box plots showing the progressively strengthened support (loop strength "-auxin" – "+auxin", obs/exp) by structural loops of cluster4 (n=1081) CRE loops. P values were computed through two-sided paired Wilcoxon signed-rank test. Boxplots present upper and lower quartiles with center line as median. Whiskers denote 1.5 × interquartile range (IQR). **e**, Box plots showing the ChIP-seq signals of indicated histone marks at anchors of cluster3 (n=744) or cluster4 (n=896) CRE loops. P values were computed through two-sided Wilcoxon signed-rank test. Boxplots present upper and lower quartiles with center line as median. Whiskers denote 1.5 × interquartile range (IQR).

Supplementary Figure 9

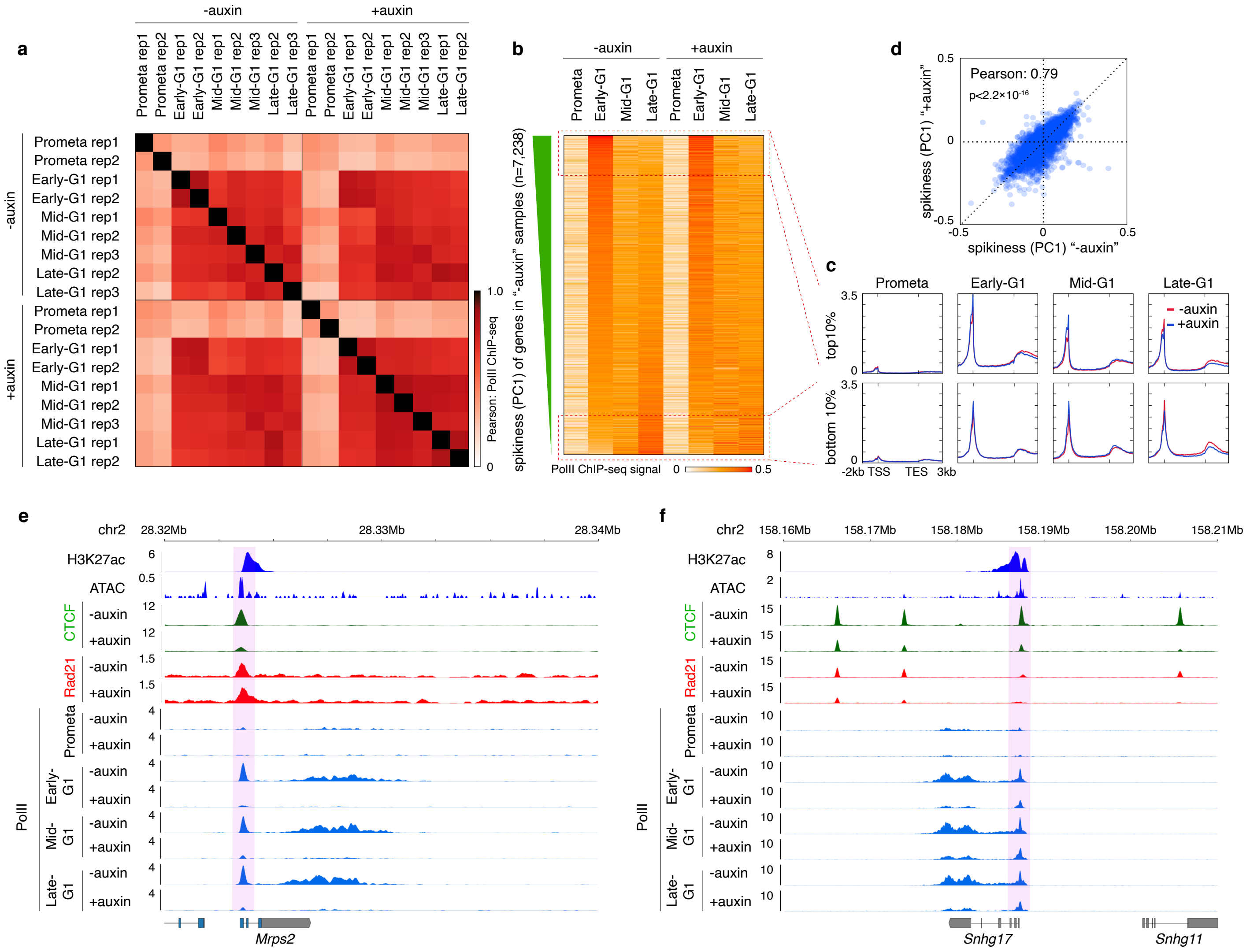

Supplementary Figure 9

a, Pearson correlations of PolII ChIP-seq signals across biological replicates. **b**, Heatmap of post-mitotic transcriptional "spikiness" of genes in untreated and auxin treated samples. Active genes were ranked in a descending order of the PC1 of untreated samples. **c**, Meta-region plots (scaled from TSS to TES) showing the PolII ChIP-seq signals of the 10% most spiking and least spiking genes across cell cycle stages with or without CTCF. **d**, Scatter plot showing the positive correlation between PC1 values of untreated and auxin treated sample, confirming that post-mitotic spiking is largely preserved after CTCF depletion. **e**, Browser tracks of the *Mrps2* locus showing dramatically reduced PolII ChIP-seq signals after CTCF depletion. Note a strong CTCF peak located at the TSS that was diminished upon auxin treatment. However, the Rad21 signal was minimally perturbed after CTCF depletion at this site, suggesting that cohesin is loaded at this region. **f**, Similar to (**e**), showing the PolII signal reduction at *Snhg17* locus.

Supplementary Figure 10

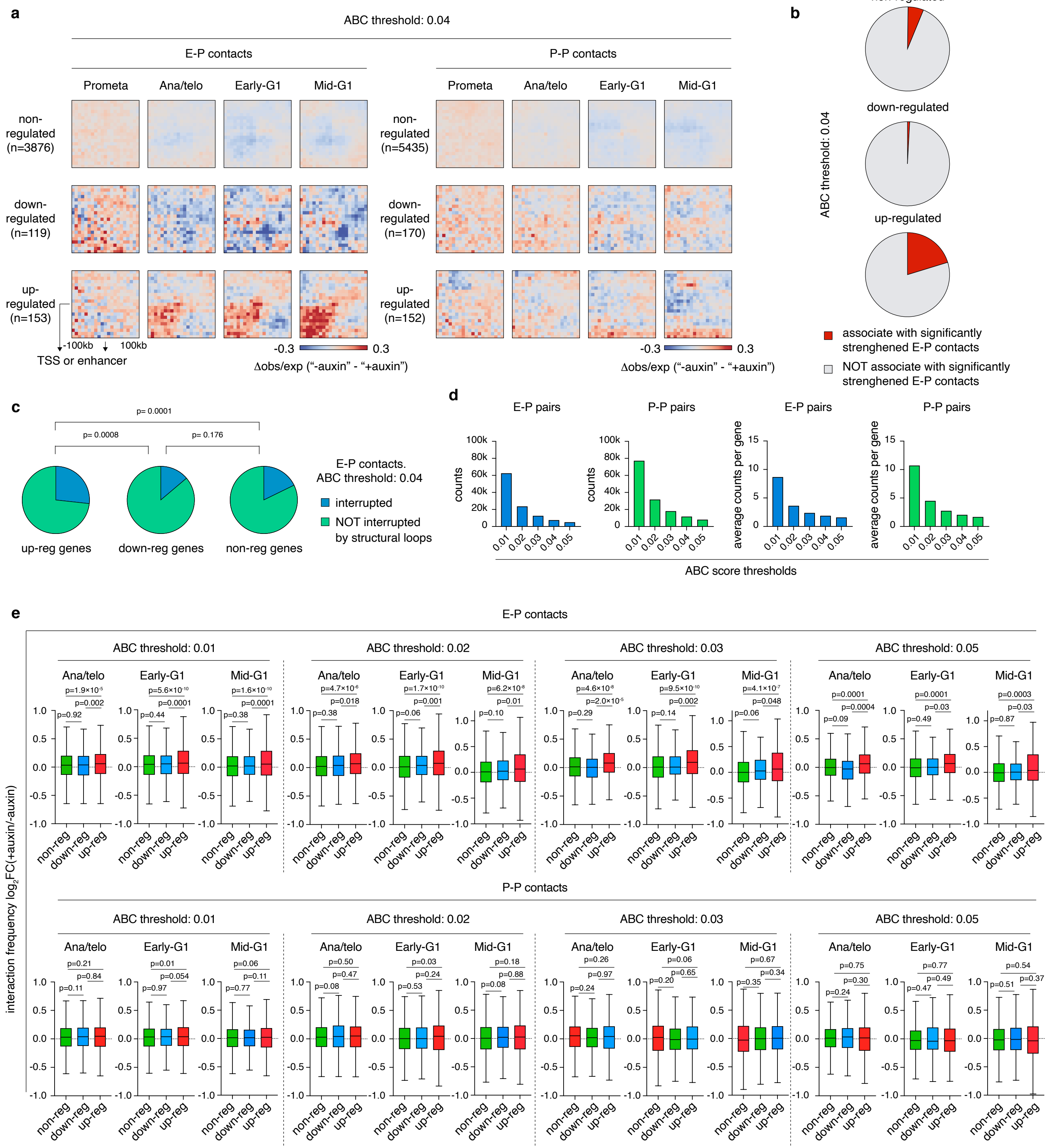

Supplementary Figure 10

a, Left: Pile-up Hi-C matrices showing the changes of E-P (enhancer-promoter) interactions associated with non-regulated, down-regulated or up-regulated genes after CTCF depletion. Bin size: 10kb. Note that increase of E-P interactions for up-regulated genes was observed as early as in ana/telophase. Right: Similar to left with pile-up analysis showing changes of P-P (promoter-promoter) interactions. Bin size: 10kb. **b**, Pie charts showing the fraction of up-, down- or non-regulated genes that are associated with significantly strengthened E-P contacts. **c**, Pie charts showing the fraction of structural loop-interrupted E-P pairs associated with non-regulated, down-regulated or up-regulated genes when ABC cutoff equals 0.04. P values were calculated using two-sided Fisher's exact test. **d**, Numbers of total and per gene confident E-P pairs or P-P pairs with ABC score cutoffs set to 0.01, 0.02, 0.03, 0.04 or 0.05. **e**, Similar to **Figure 4e-f**, with Boxplots showing the $\log_2$ fold change of interactions between E-P pairs or P-P pairs associated with non-regulated, down-regulated and up-regulated respectively with ABC scores set to 0.01 (n=58751, 1721, 2041 E-P pairs and 73141, 2108, 1982 P-P pairs for non-regulated, down-regulated and up-regulated genes respectively), 0.02 (n=22156, 653, 872 E-P pairs and 30135, 992, 824 P-P pairs for non-regulated, down-regulated and up-regulated genes respectively), 0.03 (n=11773, 336, 508 E-P pairs and 17165, 539, 464 P-P pairs for non-regulated, down-regulated and up-regulated genes respectively) or 0.05 (n=4727, 130, 219 E-P pairs and 7784, 257, 194 P-P pairs for non-regulated, down-regulated and up-regulated genes respectively). P values were computed through two-sided Wilcoxon signed-rank test. Boxplots present upper and lower quartiles with center line as median. Whiskers denote 1.5 × interquartile range (IQR).

Supplementary Figure 11

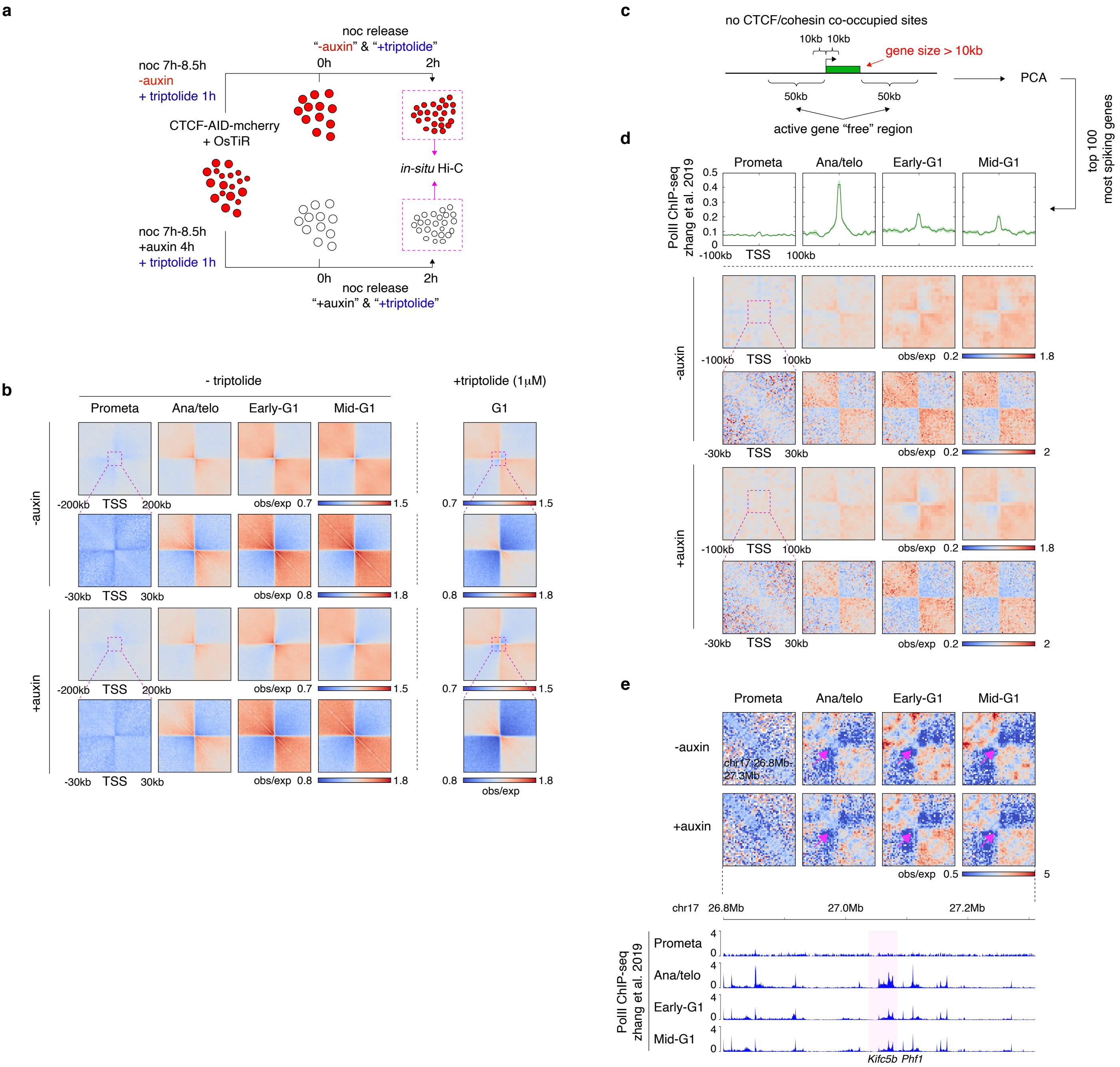

Supplementary Figure 11

a, Outline of experimental flow involving transcriptional inhibition and CTCF depletion during the mitosis-to-G1 phase transition. **b**, Pile-up Hi-C matrices showing the insulation with or without triptolide treatment at all active TSS identified previously[1]. Insulation plots were generated across cell cycle stages in both untreated control and auxin treated cells. Plots with bin size of 10kb and 1kb (zoom-in view) are shown. **c**, Strategy of target gene selection (see methods) to find genes with PolII spiking at the TSS. **d**, Upper panel: Meta-region plots showing the PolII ChIP-seq profiles (from parental G1E-ER4 cells) of the top 100 most spiking TSS chosen from (**c**). Plots were centered on TSS. Lower panel: Pile-up Hi-C matrices showing the insulation at the top 100 most spiking TSS chosen from (**c**). Plots with bin size of 10kb and 1kb (zoomed-in view) were plotted. **e**, Upper panel: KR balanced Hi-C contact matrices showing the progressive insulation gain at the *Kifc5b* and *Phf1* loci in untreated control and auxin treated samples. Insulation is indicated by purple arrows. Bin size: 10kb. Lower panel: Genome browser tracks showing PolII occupancy corresponding to genomic the region in upper panel across cell cycle stages in parental cells.

Supplementary Figure 12

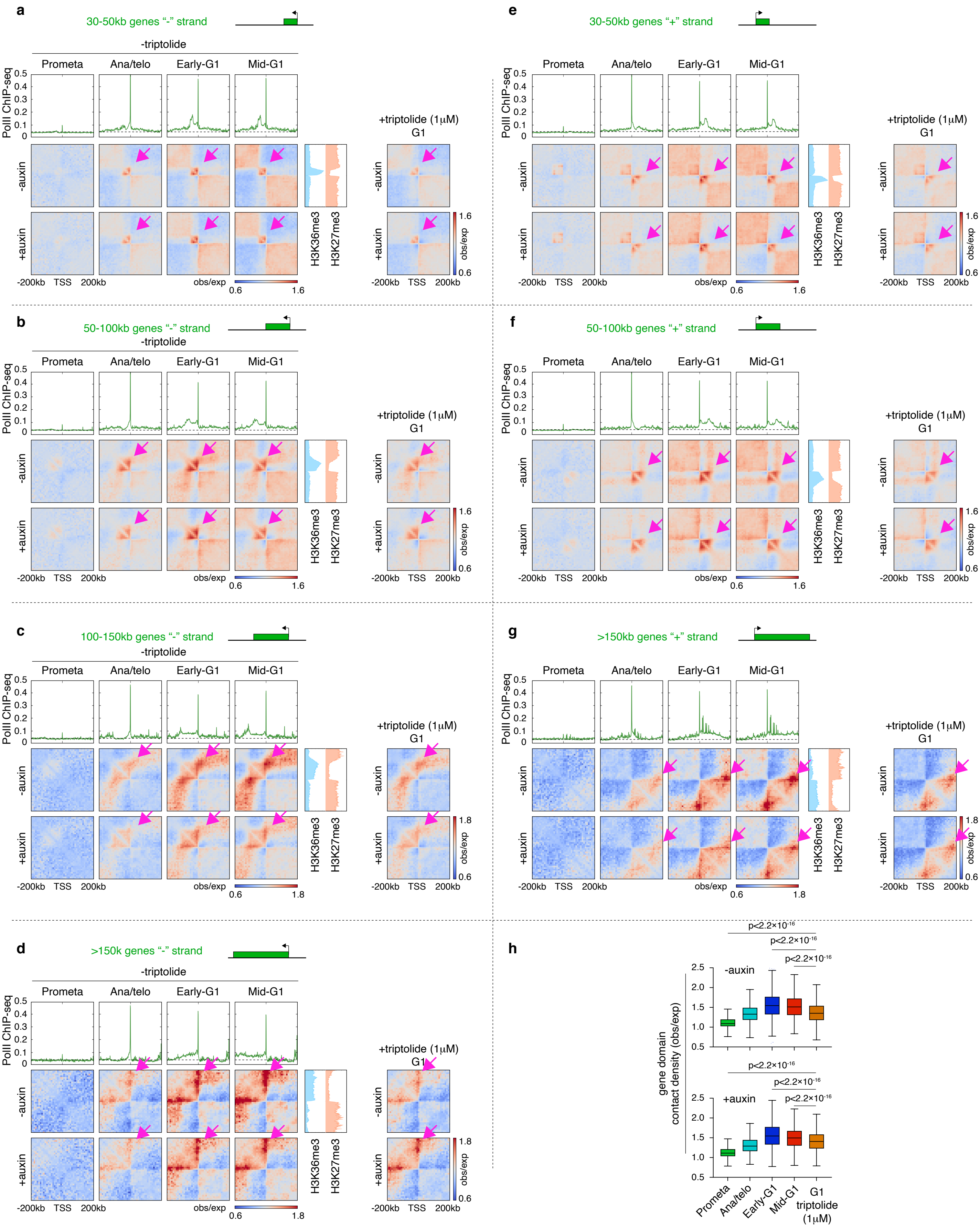

Supplementary Figure 12

a, Upper panels: Meta-region plots showing the parental PolII ChIP-seq profile of all minus strand genes between 30kb and 50kb across all cell cycle stages. Plots were centered on TSS. Bottom panels: Pile-up Hi-C matrices showing the progressive reformation of gene domains corresponding to the upper panel in samples with or without triptolide treatment. Corresponding samples after auxin treatment are also shown. Plots are centered around TSS. Gene domains are indicated by purple arrows. Meta-region plots of showing H3K36me3 and H3K27me3 ChIP-seq are sh. **b**-**g**, Similar to (**a**) showing PolII elongation and reformation of gene domains with indicated gene size range and strandedness in samples with or without triptolide treatment. Corresponding samples with auxin treatment are also shown. **h**, Box plots showing the strength of gene domains (n=2177 gene domains) in post-mitotic samples without triptolide treatment as well as G1 samples with triptolide treatment. P values were computed through two-sided paired Wilcoxon signed-rank test. Boxplots present upper and lower quartiles with center line as median. Whiskers denote 1.5 × interquartile range (IQR).
