# Supplementary material for: CTCF and transcription influence chromatin structure re-configuration after mitosis
Source: Nat Commun. 2021 Aug 27;12:5157. doi: 10.1038/s41467-021-25418-5 (PMC8397779; doi:10.1038/s41467-021-25418-5)
Supplement: Supplementary file 2 — Description of Additional Supplementary Files [file 41467_2021_25418_MOESM2_ESM.docx]

**Description of Additional Supplementary Files**

**File name: Supplementary information_zhang_et_al_07_14_21**

Description: this file contains 12 supplementary figures and their corresponding figure legends. Detailed figure captions are listed as below:

Supplementary figure1: Purification of CTCF deficient cells during M-G1 phase transition.

Supplementary figure2: Global compartment re-establishment is unperturbed by CTCF depletion after mitosis.

Supplementary figure3: CTCF depletion alters local chromatin compartmentalization.

Supplementary figure4: Characterization of boundary re-establishment after mitosis upon CTCF loss.

Supplementary figure5: Distinct responses to CTCF depletion in chromatin loop reformation after mitosis.

Supplementary figure6: Transient CRE loops are terminated with the emergence of interfering structural loops.

Supplementary figure7: Relative genomic position of structural loops determines their ability to disrupt CRE loops.

Supplementary figure8: Structural loops support the formation of interactions especially among weak CREs.

Supplementary figure9: Characterization of post-mitotic gene reactivation after CTCF depletion.

Supplementary figure10: CTCF loss-actuated post-mitotic gene up-regulation is linked to elevated enhancer-promoter interactions

Supplementary figure11: Relationship between TSS PolII binding and local insulation.

Supplementary figure12: Partial uncoupling of gene domain formation from active transcription after mitosis.

**File name: supplementary data1**

Description: Hi-C data processing statistics

**File name: supplementary data2**

Description: rGMAP boundary calls

**File name: supplementary data3**

Description: Hiccups loop calls

**File name: supplementary data4**

Description: Active genes
