# Supplementary material for: CTCF and transcription influence chromatin structure re-configuration after mitosis
Source: Nat Commun. 2021 Aug 27;12:5157. doi: 10.1038/s41467-021-25418-5 (PMC8397779; doi:10.1038/s41467-021-25418-5)
Supplement: Supplementary file 7 — Reporting Summary [file 41467_2021_25418_MOESM7_ESM.pdf]

nature research

Corresponding author(s): Haoyue Zhang, Gerd Blobel

Last updated by author(s): Jun 18, 2021

Reporting Summary

Nature Research wishes to improve the reproducibility of the work that we publish. This form provides structure for consistency and transparency in reporting. For further information on Nature Research policies, see Authors & Referees and the Editorial Policy Checklist .

Statistics

For all statistical analyses, confirm that the following items are present in the figure legend, table legend, main text, or Methods section.

n/a	Confirmed	
☐	☒	The exact sample size (*n*) for each experimental group/condition, given as a discrete number and unit of measurement
☒	☐	A statement on whether measurements were taken from distinct samples or whether the same sample was measured repeatedly
☐	☒	The statistical test(s) used AND whether they are one- or two-sided<br>*Only common tests should be described solely by name; describe more complex techniques in the Methods section.*
☒	☐	A description of all covariates tested
☐	☒	A description of any assumptions or corrections, such as tests of normality and adjustment for multiple comparisons
☐	☒	A full description of the statistical parameters including central tendency (e.g. means) or other basic estimates (e.g. regression coefficient) AND variation (e.g. standard deviation) or associated estimates of uncertainty (e.g. confidence intervals)
☐	☒	For null hypothesis testing, the test statistic (e.g. *F*, *t*, *r*) with confidence intervals, effect sizes, degrees of freedom and *P* value noted<br>*Give P values as exact values whenever suitable.*
☒	☐	For Bayesian analysis, information on the choice of priors and Markov chain Monte Carlo settings
☒	☐	For hierarchical and complex designs, identification of the appropriate level for tests and full reporting of outcomes
☐	☒	Estimates of effect sizes (e.g. Cohen's *d*, Pearson's *r*), indicating how they were calculated

Our web collection on statistics for biologists contains articles on many of the points above.

Software and code

Policy information about availability of computer code

Data collection	For high-throughput sequencing data collection, we used NextSeq Control Software v2.2.0.
Data analysis	For statistical analyses, we used R (R studio, R version 3.6.1)<br>For statistical plots, we used GraphPad Prism 8.<br>For flow chart generation, we used FlowJo 10.4.0.<br>For high throughput sequencing data processing and subsequent data analyses we used:<br>HiC-Pro (2.10.0)<br>juicer_tools (1.13.02)<br>FastQC (0.11.9)<br>Bowtie2 (2.2.9)<br>SAMtools (0.1.19)<br>macs2 (2.1.0)<br>UCSC Toolkit ("bedGraphToBigWig")<br>BEDtools (2.27.1)<br>deeptools (2.5.4)<br>rGMAP (1.4)<br>hic2cool (0.8.0)<br>coolpup (0.9.2)<br>plotpup (0.9.2)<br>pygenometracks (3.1)<br>ABC enhancer prediction model: https://github.com/broadinstitute/ABC-Enhancer-Gene-Prediction<br>DESEQ2 (1.24.0)<br>Limma (3.40.6)

prcomp (3.6.1)

For manuscripts utilizing custom algorithms or software that are central to the research but not yet described in published literature, software must be made available to editors/reviewers. We strongly encourage code deposition in a community repository (e.g. GitHub). See the Nature Research guidelines for submitting code & software for further information.

Data

Policy information about availability of data

All manuscripts must include a data availability statement. This statement should provide the following information, where applicable:

- Accession codes, unique identifiers, or web links for publicly available datasets
- A list of figures that have associated raw data
- A description of any restrictions on data availability

All figures include publicly available data. Raw and processed HiC and PolII ChIP-seq data generated in this study are deposited into GEO database with accession number GSE168251 [https://www.ncbi.nlm.nih.gov/geo/query/acc.cgi?acc=GSE168251] for public access. Boundaries, loops and active genes identified in this study are provided in the supplementary information files.

Additional external ChIP–seq data of histone modifications on asynchronous cells are available at: H3K27ac (GSE61349) [https://www.ncbi.nlm.nih.gov/geo/query/acc.cgi?acc=GSE61349] , H3K4me1 (GSM946535) [https://www.ncbi.nlm.nih.gov/geo/query/acc.cgi?acc=GSM946535] , H3K4me3 (GSM946533) [https://www.ncbi.nlm.nih.gov/geo/query/acc.cgi?acc=GSM946533] , H3K36me3 (GSM946529) [https://www.ncbi.nlm.nih.gov/geo/query/acc.cgi?acc=GSM946529] 52,H3K27me3 (GSM946531) [https://www.ncbi.nlm.nih.gov/geo/query/acc.cgi?acc=GSM946531] , H3K9me3 (GSM946542) [https://www.ncbi.nlm.nih.gov/geo/query/acc.cgi?acc=GSM946542] . Additional external ChIP-seq data of CTCF, Rad21 and PolII for WT post-mitotic time points are available at GSE129997 [https://www.ncbi.nlm.nih.gov/geo/query/acc.cgi?acc=GSE129997]. External data of CTCF and Rad21 before and after CTCF depletion in asynchronous cells are available at GSE150418 [https://www.ncbi.nlm.nih.gov/geo/query/acc.cgi?acc=GSE150418] .

Field-specific reporting

Please select the one below that is the best fit for your research. If you are not sure, read the appropriate sections before making your selection.

☒ Life sciences ☐ Behavioural & social sciences ☐ Ecological, evolutionary & environmental sciences

For a reference copy of the document with all sections, see nature.com/documents/nr-reporting-summary-flat.pdf

Life sciences study design

All studies must disclose on these points even when the disclosure is negative.

Sample size	Sample size was not pre-determined. We used sample sizes commonly accepted for high throughput genome wide experiments by us and others (Zhang et al. Nature 2019, https://doi.org/10.1038/s41586-019-1778-y; Nora et al. Cell 2017, http://dx.doi.org/10.1016/j.cell.2017.05.004;). We performed 2-3 biological replicates for Hi-C, 2-3 biological replicates for ChIP-seq. Hi-C and ChIP-seq data were pooled for down-stream analyses based on overall high correlation
Data exclusions	Experiments were done in multiple replicates. One replicate of late G1 phase RNA PolII chip-seq was removed because of technical error.
Replication	2-3 biological replicates for Hi-C and 2-3 biological replicates for ChIP-seq were generated. All attempts were successful except one replicate of late G1 phase RNA PolII chip-seq was removed because of technical error. All findings in this study is reproducible among biological replicates
Randomization	Randomization is irrelevant to this study as no animal or human subjects were involved in this study.
Blinding	Blinding was not relevant to our study as no human or animal subjects were involved.

Reporting for specific materials, systems and methods

We require information from authors about some types of materials, experimental systems and methods used in many studies. Here, indicate whether each material, system or method listed is relevant to your study. If you are not sure if a list item applies to your research, read the appropriate section before selecting a response.

Materials & experimental systems

n/a	Involved in the study	
☐	☒	Antibodies
☐	☒	Eukaryotic cell lines
☒	☐	Palaeontology
☒	☐	Animals and other organisms
☒	☐	Human research participants
☒	☐	Clinical data

Methods

n/a	Involved in the study	
☐	☒	ChIP-seq
☐	☒	Flow cytometry
☒	☐	MRI-based neuroimaging

Antibodies

Antibodies used	anti-pMPM2 Millipore, catlog#: 05-368, Clone: MPM-2, multiple lots of antibodies were used. Dilution: 0.2ul/10million cells<br>anti-Pol II Cell Signaling, catlog#: 14958, Clone: D8L4Y, lot# 1. Dilution: 5ug/IP<br>F(ab')2-goat anti-mouse secondary antibody, APC, Thermo Fisher Scientific, catelog#: 17-4010-82. Polyclonal, lot# 1997054. Dilution: 20ul/10million cells.
Validation	anti-pMPM2 Millipore, catlog#: 05-368, multiple lots of antibodies were used. This antibody has been claimed to react with mouse pMPM2 by the manufacturer. This antibody has been previously used in our lab (Behera et al. 2019, Cell Reports, Zhang et al. 2019 Nature, Hsiung et al. 2016 Genes & Development)<br><br>anti-Pol II Cell Signaling, 14958, lot# 1. This antibody has been claimed to react with mouse and to be suitable for ChIP by the manufacture. This antibody has also been previously used in our lab (Behera et al. 2019, Cell Reports, Zhang et al. 2019 Nature) and also by others for ChIP experiments (eg. Sun Y. et al. 2019, Science Advances.)<br><br>F(ab')2-goat anti-mouse secondary antibody, APC, Thermo Fisher Scientific, catelog#: 17-4010-82. lot# 1997054. The manufacturer has tested this antibody to be suitable for immunofluorescence studies.

Eukaryotic cell lines

Policy information about cell lines

Cell line source(s)	The G1E-ER4 cell line was a gift from Mitchell Weiss' laboratory.
Authentication	We regularly confirm that these cells can be induced to undergo terminal erythroid differentiation through both visual inspection and RT-qPCR using primers targeting murine b-globin gene transcripts.
Mycoplasma contamination	G1E-ER4 cells has been tested to be negative of Mycoplasma
Commonly misidentified lines<br>(See ICLAC register)	The cell line used (G1E-ER4) is not in the ICLAC database

ChIP-seq

Data deposition

☐ Confirm that both raw and final processed data have been deposited in a public database such as GEO.

☒ Confirm that you have deposited or provided access to graph files (e.g. BED files) for the called peaks.

Data access links<br>*May remain private before publication.*	https://www.ncbi.nlm.nih.gov/geo/query/acc.cgi?acc=GSE168251
Files in database submission	run236_2183_Read1.fastq.gz<br>run250_2299_Read1.fastq.gz<br>run236_2186_Read1.fastq.gz<br>run250_2303_Read1.fastq.gz<br>run236_2184_Read1.fastq.gz<br>run250_2300_Read1.fastq.gz<br>run236_2187_Read1.fastq.gz<br>run250_2304_Read1.fastq.gz<br>run236_2185_Read1.fastq.gz<br>run250_2301_Read1.fastq.gz<br>run236_2188_Read1.fastq.gz<br>run250_2305_Read1.fastq.gz<br>run180_1714_Read1.fastq.gz

run250_2302_Read1.fastq.gz
run180_1713_Read1.fastq.gz
run180_1716_Read1.fastq.gz
run250_2306_Read1.fastq.gz
run180_1715_Read1.fastq.gz
2929.run351_Read1.fastq.gz
2930.run351_Read1.fastq.gz
2931.run351_Read1.fastq.gz
2932.run351_Read1.fastq.gz
run236_2183_Read2.fastq.gz
run250_2299_Read2.fastq.gz
run236_2186_Read2.fastq.gz
run250_2303_Read2.fastq.gz
run236_2184_Read2.fastq.gz
run250_2300_Read2.fastq.gz
run236_2187_Read2.fastq.gz
run250_2304_Read2.fastq.gz
run236_2185_Read2.fastq.gz
run250_2301_Read2.fastq.gz
run236_2188_Read2.fastq.gz
run250_2305_Read2.fastq.gz
run180_1714_Read2.fastq.gz
run250_2302_Read2.fastq.gz
run180_1713_Read2.fastq.gz
run180_1716_Read2.fastq.gz
run250_2306_Read2.fastq.gz
run180_1715_Read2.fastq.gz
2929.run351_Read2.fastq.gz
2930.run351_Read2.fastq.gz
2931.run351_Read2.fastq.gz
2932.run351_Read2.fastq.gz
run240_2183_Read1.fastq.gz
run251_2299_Read1.fastq.gz
run240_2186_Read1.fastq.gz
run251_2303_Read1.fastq.gz
run240_2184_Read1.fastq.gz
run251_2300_Read1.fastq.gz
run240_2187_Read1.fastq.gz
run251_2304_Read1.fastq.gz
run240_2185_Read1.fastq.gz
run251_2301_Read1.fastq.gz
run240_2188_Read1.fastq.gz
run251_2305_Read1.fastq.gz
run240_1714_Read1.fastq.gz
run251_2302_Read1.fastq.gz
run240_1716_Read1.fastq.gz
run251_2306_Read1.fastq.gz
2929.run352_Read1.fastq.gz
2930.run352_Read1.fastq.gz
2931.run352_Read1.fastq.gz
2932.run352_Read1.fastq.gz
run240_2183_Read2.fastq.gz
run251_2299_Read2.fastq.gz
run240_2186_Read2.fastq.gz
run251_2303_Read2.fastq.gz
run240_2184_Read2.fastq.gz
run251_2300_Read2.fastq.gz
run240_2187_Read2.fastq.gz
run251_2304_Read2.fastq.gz
run240_2185_Read2.fastq.gz
run251_2301_Read2.fastq.gz
run240_2188_Read2.fastq.gz
run251_2305_Read2.fastq.gz
run240_1714_Read2.fastq.gz
run251_2302_Read2.fastq.gz
run240_1716_Read2.fastq.gz
run251_2306_Read2.fastq.gz
2929.run352_Read2.fastq.gz

2930.run352_Read2.fastq.gz
2931.run352_Read2.fastq.gz
2932.run352_Read2.fastq.gz
run241_2183_Read1.fastq.gz
run241_2186_Read1.fastq.gz
run253_2303_Read1.fastq.gz
run241_2184_Read1.fastq.gz
run253_2300_Read1.fastq.gz
run241_2187_Read1.fastq.gz
run253_2304_Read1.fastq.gz
run241_2185_Read1.fastq.gz
run253_2301_Read1.fastq.gz
run241_2188_Read1.fastq.gz
run253_2305_Read1.fastq.gz
run253_2302_Read1.fastq.gz
2929.run353_Read1.fastq.gz
2930.run353_Read1.fastq.gz
2931.run353_Read1.fastq.gz
2932.run353_Read1.fastq.gz
run241_2183_Read2.fastq.gz
run241_2186_Read2.fastq.gz
run253_2303_Read2.fastq.gz
run241_2184_Read2.fastq.gz
run253_2300_Read2.fastq.gz
run241_2187_Read2.fastq.gz
run253_2304_Read2.fastq.gz
run241_2185_Read2.fastq.gz
run253_2301_Read2.fastq.gz
run241_2188_Read2.fastq.gz
run253_2305_Read2.fastq.gz
run253_2302_Read2.fastq.gz
2929.run353_Read2.fastq.gz
2930.run353_Read2.fastq.gz
2931.run353_Read2.fastq.gz
2932.run353_Read2.fastq.gz
0h_no_a_rep1.cool
0h_no_a_rep2.cool
0h_with_a_rep1.cool
0h_with_a_rep2.cool
30min_no_a_rep1.cool
30min_no_a_rep2.cool
30min_with_a_rep1.cool
30min_with_a_rep2.cool
60min_no_a_rep1.cool
60min_no_a_rep2.cool
60min_with_a_rep1.cool
60min_with_a_rep2.cool
120min_no_a_rep1.cool
120min_no_a_rep2.cool
120min_no_a_rep3.cool
120min_with_a_rep1.cool
120min_with_a_rep2.cool
120min_with_a_rep3.cool
120min_no_a_with_trip_rep1.cool
120min_no_a_with_trip_rep2.cool
120min_with_a_with_trip_rep1.cool
120min_with_a_with_trip_rep2.cool
boundary_calls.csv
loop_calls.csv
PolII-0h-A.rep1.fastq.gz
PolII-0h-A.rep2.fastq.gz
PolII-0h+A.rep1.fastq.gz
PolII-0h+A.rep2.fastq.gz
PolII-1h-A.rep1.fastq.gz
PolII-1h-A.rep2.fastq.gz
PolII-1h+A.rep1.fastq.gz
PolII-1h+A.rep2.fastq.gz
PolII-2h-A.rep1.fastq.gz

PolII-2h-A.rep2.fastq.gz
PolII-2h-A.rep3.fastq.gz
PolII-2h+A.rep1.fastq.gz
PolII-2h+A.rep2.fastq.gz
PolII-2h+A.rep3.fastq.gz
PolII-4h-A.rep2.fastq.gz
PolII-4h-A.rep3.fastq.gz
PolII-4h+A.rep1.fastq.gz
PolII-4h+A.rep2.fastq.gz
Input-Noc0h.fastq.gz
Input-Noc1h.fastq.gz
Input-Noc2h.fastq.gz
Input-Noc4h.fastq.gz
PolII-0h-A.rep1.bw
PolII-0h+A.rep1.bw
PolII-0h-A.rep2.bw
PolII-0h+A.rep2.bw
PolII-1h-A.rep1.bw
PolII-1h+A.rep1.bw
PolII-1h-A.rep2.bw
PolII-1h+A.rep2.bw
PolII-2h-A.rep1.bw
PolII-2h+A.rep1.bw
PolII-2h-A.rep2.bw
PolII-2h+A.rep2.bw
PolII-2h-A.rep3.bw
PolII-2h+A.rep3.bw
PolII-4h+A.rep1.bw
PolII-4h-A.rep2.bw
PolII-4h+A.rep2.bw
PolII-4h-A.rep3.bw
PolII-0h-A.rep1.narrowPeak
PolII-0h+A.rep1.narrowPeak
PolII-0h-A.rep2.narrowPeak
PolII-0h+A.rep2.narrowPeak
PolII-1h-A.rep1.narrowPeak
PolII-1h+A.rep1.narrowPeak
PolII-1h-A.rep2.narrowPeak
PolII-1h+A.rep2.narrowPeak
PolII-2h-A.rep1.narrowPeak
PolII-2h+A.rep1.narrowPeak
PolII-2h-A.rep2.narrowPeak
PolII-2h+A.rep2.narrowPeak
PolII-2h-A.rep3.narrowPeak
PolII-2h+A.rep3.narrowPeak
PolII-4h+A.rep1.narrowPeak
PolII-4h-A.rep2.narrowPeak
PolII-4h+A.rep2.narrowPeak
PolII-4h-A.rep3.narrowPeak

Genome browser session
(e.g. UCSC)

https://genome.ucsc.edu/s/yeminlan/Adam_PolII_ChIPseq

Methodology

Replicates

See methods section. Briefly, 2-3 biological replicates were generated per time point per auxin treatment condition Specifically:

For Pol II, we performed 3 biological replicates for mid-G1 phase (+/-auxin) and 2 biological replicates for other time points (prometa, early-G1 and late-G1, +/-auxin)

Sequencing depth

total reads uniquely mapped reads aligned >1 times overall alignment rate length single or paired
PolII-0h-A 26730558 11430951 10742968 82.95% 50 single-end
PolII-0h+A 26752788 11147007 10430002 80.65% 50 single-end
PolII-0h-A.rep2 31949509 11832634 11736626 73.77% 50 single-end
PolII-0h+A.rep2 27776259 11235653 10435263 78.02% 50 single-end

PolII-1h-A 27327682 15208305 10138826 92.75% 50 single-end
PolII-1h+A 23453572 12986043 8448516 91.39% 50 single-end
PolII-1h-A.rep2 35424605 19350113 13657313 93.18% 50 single-end
PolII-1h+A.rep2 31092227 16690936 11384586 90.30% 50 single-end
PolII-2h-A 20404953 10208768 7498640 86.78% 50 single-end
PolII-2h+A 25789763 14215212 9548641 92.14% 50 single-end
PolII-2h-A.rep2 31897226 17003062 12380556 92.12% 50 single-end
PolII-2h+A.rep2 31515363 16940756 11215688 89.34% 50 single-end
PolII-2h-A.rep3 52784121 24697293 17568125 80.07% 50 single-end
PolII-2h+A.rep3 52774419 22931393 18547164 78.60% 50 single-end
PolII-4h+A 29982588 15997733 11205426 90.73% 50 single-end
PolII-4h-A.rep2 28984552 15422595 11606712 93.25% 50 single-end
PolII-4h+A.rep2 36309391 16513948 12727278 80.53% 50 single-end
PolII-4h-A.rep3 47893566 24778021 16551382 86.29% 50 single-end

Antibodies

anti-Pol II Cell Signaling, 14958

Peak calling parameters

Sequencing reads were mapped to mm9 using Bowtie2 (v2.2.9) with default parameters. Aligments with MAPQ score smaller than 10 were discarded. Duplicates were removed using Samtools (v0.1.19), and in addition, reads on mitochondria, contigs and blacklist regions were removed. BAM files were converted to BED files using Bedtools (v2.27.1). Peaks were called using MACS2 (v2.1.0) over corresponding input controls, with default parameters and 0.01 q-value cutoff.

Data quality

(1). Raw fastq files were assessed with FastQC (v0.11.5) prior to processing.
(2). Peaks were called using input controls corresponding to every cell cycle stage.
(3). Peaks were called using p-value cutoffs described above and aligments with MAPQ score smaller than 10 were discarded
(4). Correlation among replicates was assessed (Extended Data Fig. 8a).
(5). Peaks of Pol II were largely ocated at expected genomic regions (TSS, genebodies).

Software

For ChIP-seq data processing and analyses, we used Bowtie2 (v2.2.9), Samtools v0.1.19, MACS2 (v2.1.0), kent UCSC Utilities, bwtool version 1.0, BEDtools,2.27.1 and deeptools 2.5.4

Flow Cytometry

Plots

Confirm that:

- [x] The axis labels state the marker and fluorochrome used (e.g. CD4-FITC).
- [x] The axis scales are clearly visible. Include numbers along axes only for bottom left plot of group (a 'group' is an analysis of identical markers).
- [x] All plots are contour plots with outliers or pseudocolor plots.
- [ ] A numerical value for number of cells or percentage (with statistics) is provided.

Methodology

Sample preparation

See method section.
Briefly, actively proliferating G1E-ER4 cells were synchronized at pro-metaphase with nocodazole treatment. Samples were added with auxin to deplete CTCF for 4h during synchronization. Cells were then released from nocodazole for several durations with or without auxin treatment (0h, 30min, 1h and 2h). For transcription inhibition experiments, triptolide were added into the culture for 1h during synchronization and triptolide was maintained after nocodazole release for 2h before harvest. Cells were harvested, washed with PBS and crosslinked with 1% PFA for 10min. Crosslinks were quenched with glycine for 5min and cells were then penetrated with 0.1% Triton X-100. Finally, cells were stained with 20ng/ml DAPI and subjected to cell sorting.

For prometaphase cells (0h), cells were stained with pMPM2 antibody and APC-conjugated F(ab')2-goat anti-mouse secondary antibody for mitotic sorting

Instrument

Beckman Coulter Moflo Astrios sorter/Becton Dickinson FACSAria Fusion sorter

Software

Flow charts were generated using FlowJo 10.4.0

Cell population abundance

for Hi-C and ChIP-seq experiments. 7-10million cells were collected for G1 phases and 4-5 million cells were collected for prometaphase and ana/telophase

Gating strategy	Prometaphase cells were gated on mcherry (low for auxin treated samples and high for untreated control samples), DAPI (4N) and pMPM2 (high) fluorescent signal.<br><br>ana/telophase cells were gated based on DAPI (4N), GFP (reduced) and mcherry (low for auxin treated samples and high for untreated control samples) signals. G1 samples were gated based on DAPI (2N) and mcherry (low for auxin treated samples and high for untreated control samples) signals.

☒ Tick this box to confirm that a figure exemplifying the gating strategy is provided in the Supplementary Information.
